# Asthma diagnosis and treatment – 1008. Is small airways disease a widely prevalent yet underdiagnosed phenotype of asthma and COPD in India?

**DOI:** 10.1186/1939-4551-6-S1-P8

**Published:** 2013-04-23

**Authors:** Sandeep Tandon, Arvind Khutarkar, Shabina Ansari

**Affiliations:** 1 Pulmonary Medical Unit, Tata Memorial Hospital, Mumbai, India; 2 Staff Clinic, Tata Memorial Hospital, Mumbai, India

## Background

Spirometry reports of many patients with clinical features of Obstructive Airways Disease (OAD) show predominant small airways(SAW) disease with reduced flow rates, but GOLD or GINA spirometric criteria not diagnostic of COPD or Asthma.

## Aims

To compare small airways spirometric parameters with GOLD/GINA diagnostic parameters for COPD/Asthma in clinically and FlowVolume(FV) Loop-wise suspected OAD.

## Methods

We retrospectively reviewed clinical data and spirometry reports of patients referred for preoperative respiratory fitness. We reviewed reports of patients whose histories, clinical findings and Flow-Volume Loop obstructive patterns were consistent with COPD/Asthma, but which were not diagnostic of COPD/Asthma based on the GOLD/GINA criteria. Data was analysed for 49 patients.

## Results

Data comprised 14 males, 35 females, mean age 54yrs (31-72), 82 % never-smokers. 44 had post bronchodilator studies. FEF75 or FEF25-75 < 65% were categorised as SAW disease. Pre bronchodilator (Pre-BD) Mean FEV1/FVC was 78% (70-91%), however Mean FEF75 and FEF25-75 (% predicted) were 34.5% and 43.3% respectively. 6 patients had both, a reversibility in FEV1 >12% and increase by 200ml. Interestingly, of the remaining 38 patients who had no reversibility on a postbronchodilator study, 19 had > 30% reversibility in either FEF 25-75 or FEF75 (Mean reversibility of 53.5%) with a mean post bronchodilator increase in flow rates of 506.3 mL/s. All patients improved symptomatically with inhaled bronchodilator ± steroid therapy.

## Conclusions

Small airways (SAW) disease is an important feature of both COPD and Asthma. Screening spirometry relying only on FEV1, FVC, FEV6 without small airways flow rates or visually documented obstructive pattern on FV Loops may miss SAW disease, thus underdiagnosing such phenotypes of COPD or Asthma which might be prevalent in Asian countries due to exposure to biomass fuel and air pollution.

